# Use of a draft genome of coffee (C*offea arabica*) to identify SNPs associated with caffeine content

**DOI:** 10.1111/pbi.12912

**Published:** 2018-04-13

**Authors:** Hue T.M. Tran, Thiruvarangan Ramaraj, Agnelo Furtado, Leonard Slade Lee, Robert J. Henry

**Affiliations:** ^1^ Queensland Alliance for Agriculture and Food Innovation (QAAFI) The University of Queensland St Lucia Qld Australia; ^2^ Western Highlands Agriculture & Forestry Science Institute (WASI) Buon Ma Thuot Vietnam; ^3^ National Center for Genome Resources (NCGR) Santa Fe NM USA

**Keywords:** Arabica coffee, genome assembly, genome annotation, caffeine, single nucleotide polymorphism discovery, association

## Abstract

Arabica coffee (*Coffea arabica)* has a small gene pool limiting genetic improvement. Selection for caffeine content within this gene pool would be assisted by identification of the genes controlling this important trait. Sequencing of DNA bulks from 18 genotypes with extreme high‐ or low‐caffeine content from a population of 232 genotypes was used to identify linked polymorphisms. To obtain a reference genome, a whole genome assembly of arabica coffee (variety K7) was achieved by sequencing using short read (Illumina) and long‐read (PacBio) technology. Assembly was performed using a range of assembly tools resulting in 76 409 scaffolds with a scaffold N50 of 54 544 bp and a total scaffold length of 1448 Mb. Validation of the genome assembly using different tools showed high completeness of the genome. More than 99% of transcriptome sequences mapped to the *C. arabica* draft genome, and 89% of BUSCOs were present. The assembled genome annotated using AUGUSTUS yielded 99 829 gene models. Using the draft arabica genome as reference in mapping and variant calling allowed the detection of 1444 nonsynonymous single nucleotide polymorphisms (SNPs) associated with caffeine content. Based on Kyoto Encyclopaedia of Genes and Genomes pathway‐based analysis, 65 caffeine‐associated SNPs were discovered, among which 11 SNPs were associated with genes encoding enzymes involved in the conversion of substrates, which participate in the caffeine biosynthesis pathways. This analysis demonstrated the complex genetic control of this key trait in coffee.

## Introduction

Coffee is an important crop, and world coffee production relies on only two species, *Coffea canephora* (robusta) and *C. arabica* (arabica), of which arabica is the dominant species and thus has a higher priority for genetic improvement. The chemical composition of coffee bean comprising nonvolatile and volatile compounds plays a decisive role in coffee quality. Among the nonvolatiles, caffeine is one of the most important compounds contributing to the strength, body and bitterness of brewed coffee (Trugo, 1984). Genes involved in the metabolism of caffeine have been widely studied and described (Ashihara, 2006; Ogawa *et al*., 2001; Ogita *et al*., 2003, 2004; Salmona *et al*., 2008; Uefuji *et al*., 2003). However, the caffeine biosynthesis genes were identified based on sequences derived from a limited number of arabica cultivars. The identification of single nucleotide polymorphism (SNPs) associated with bean caffeine content in diverse populations will potentially help support the manipulation of this compound in arabica coffee utilizing molecular markers. While low caffeine or zero caffeine attracts the attention of some coffee customers, with about a 10% share of the global market, but 16% in the United States, 13% in the UK and 17% in Spain (ITC, 2009), the manipulation of caffeine content would be very significant for the coffee industry.

An available and reliable reference genome is vital for the identification of genetic variations (SNPs or Indels) in genes and their regulatory sequences and supporting genomewide association studies, genomic selection and transgenic applications (summarized by Margarido and Heckerman, 2015). Recently, the first high‐quality draft genome of robusta coffee was completed (Denoeud *et al*., 2014). This genome sequence provides a reference for analysis of the genomes of other *Coffea* species and genotypes. However, *C. canephora* is a diploid species while *C. arabica* is tetraploid. Obtaining a *C. arabica* whole genome sequence will provide a much better platform for arabica coffee genetics.

This report describes genome assembly, validation and annotation, followed by the identification of SNPs associated with caffeine using the draft genome as reference.

## Results

### Sequencing details

A total of 385 million reads of 100‐bp Illumina pair end (PE) estimated at 64× coverage (insert size of 350 bp) and 220.10^6^ reads (equivalent to 36×) and 223.10^6^ reads (equivalent to 37× coverage) of 100‐bp Illumina mate pair (MP) of insert size of 3 and 8 kb, respectively, were generated on an Illumina HiSeq 2000. Over 1.37 million PacBio reads, equivalent to 6× genome coverage, were also generated using the SMRT P6‐C4 chemistry (Table S1).

The coverage of Illumina data was 137× (both PE and MP), while that of PacBio was only 6×. The GC content in K7 arabica variety was from 37% to 40%. The Phred score for both paired‐end and MP was 39 (Table S1). These data sets were the input sequence used for hybrid assembly of *C. arabica*.

### Genome assembly

#### Comparison among different genome assemblers using Illumina data

Genome assembly was performed using different software programs including CLC Genomics Workbench (GWB), ABySS, PLATANUS and SOAP*denovo*2. The assembly outputs of these four assemblers are provided in Table S2.

The CLC GWB was applied to both PE and MP reads assembly, yielding a total length of 800 884 967 bp (61.61% of the genome size) and a fairly good N50 value (5490 bp) (Table S2). After the second step of scaffolding, ABySS yielded a reasonably high (8987 bp) scaffold N50. However, the total genome length without gaps only covered 38.5% of the estimated genome of arabica. The number of scaffolds and scaffold N50 in PLATANUS was slightly better than others; however, the total genome length was small, only covering 20.6% of the expected genome size (Table S2). SOAP*denovo*2 outperformed other assemblers with a total genome length (without gaps) of 813 528 413 bp covering 62.6% of the estimated genome size of 1.3 Gb even though the scaffold N50 (16 694 bp) was slightly lower than for PLATANUS.

#### Assembly improvement using Illumina and PacBio reads

GAPCloser (a module in SOAP) was deployed to address some of the gaps (N's) emerging from scaffolding steps in SOAP*denovo*2. SSPACE Standard (Boetzer *et al*., 2011) was then used for scaffolding, followed by GAPCloser again to fill N regions in the scaffolds using Illumina PE and MP reads. Basic assembly metrics (i.e. contig N50, scaffold N50, total genome length, the total gap length) were improved after each step of gap filling and scaffolding (Table S3). The addition of PacBio data, even with low coverage, showed improvement in terms of assembly metrics and resulted in the draft genome as presented in Table 1.

**Table 1:** Characteristics of the K7 arabica draft genome assembly

Estimated genome size (Mb)	1300
Chromosome number (2n = 4x)	44
Total size of assembled contigs (Mb)	1167
Number of contigs	265 687
Largest contig (bp)	186 701
N50 length (contigs) (bp)	12 184
Number of scaffolds	76 409
Total size of assembled scaffolds (Mb)	1448
N50 length (scaffolds) (bp)	54 544
Longest scaffold (bp)	769 411
Number of gaps	189 278
Mean gaps length (bp)	1485
Total size of gaps (Mb)	281
GC content (%)	37

The total size of the assembled contigs was 1167 Mb, which was 90% of the estimated genome size (i.e. 1300 Mb), while that of scaffolds was 1448 Mb—11% larger than the estimated genome (Table 1). The draft genome included 76 409 scaffolds with N50 scaffolds of 54 544 bp and longest scaffold of 769 411 bp. The draft genome had a total size of gaps of 281 039 881 bp, accounting for 21% of the estimated genome.

#### Validation of genome assembly

Illumina short reads (PE) were aligned back to the assembled genome using BWA to evaluate the genome completeness and to detect errors in the assembly. More than 98% of the short reads mapped to the genome, and more than 93% were marked as properly paired (Table 2).

**Table 2:** Validation of draft genome using BWA, GMAP and BUSCO

Results of read remapping using BWA	
Read alignment metrics	
Total number of reads mapping back	98.4%
Reads properly paired	93.0%

Gene capture analysis using GMAP (*Coffea canephora* CDS sequences)		
Parameters	CDS sequences in *C. canephora*	Transcriptome of *C. arabica* (K7)
Total number of sequences	25 574	96 521
Total number of sequences mapping to *C. arabica* (draft genome)	>99.0%	>99.0%
Total number of sequences mapping to *C. arabica* (draft genome) (≥90% identity and 90% query coverage)	>85.0%	>88.0%

BUSCO analysis		
Parameters	Number of BUSCOs mapped	Percentage
Complete single‐copy BUSCOs (C)	858	89%
Complete duplicated BUSCOs [D]	553	57%
Fragmented BUSCOs (F)	23	2.4%
Missing BUSCOs (M)	75	7.8%
Total BUSCO groups searched (*n*)	956	

The *C. canephora* CDS sequences and *C. arabica* (K7) PacBio transcriptome data were used to map back to the draft genome using GMAP. More than 99% of the CDS and transcriptome sequences were mapped to the *C. arabica* draft genome in which 85.1% of the CDS sequences and 88% of transcriptome sequences with ≥90% identity and query coverage were mapped (Table 2).

BUSCO analysis against plant‐specific database of 956 genes was also used to assess the completeness of the draft genome and identified 858 (89%) complete BUSCOs, of which 553 (57%) were duplicated. A further 23 fragmented BUSCOs were identified (Table 2).

#### Genome annotation

The gene prediction programs, SNAP and AUGUSTUS, were used with tomato genome as reference. ESTs of *C. arabica* and CDSs of *C. canephora* and 96 521 in‐house *C. arabica* (K7) PacBio transcriptome sequences were used as evidence to guide the annotation process. Altogether, 24 478 gene models were predicted consistently with different parameters when using MAKER. When performed with SNAP and AUGUSTUS, the number of gene models reached to 99 829 using tomato as reference. Mean length of gene was 2612 bp with min length as low as 49 bp to max length of up to 51 554 bp (Table S5).

#### Mapping and SNP identification

Sequencing of each DNA bulk resulted in more than 230 and 320 million high‐quality reads with the coverage (depth) of 28 and 40× for the low and high‐caffeine bulks, respectively (Table 3). The GC content of this study was 36%, the Phred score was 37, and the average length after trimming was 147 bp for both bulks. The number of variants called between the two bulks (18 469) was dramatically reduced compared to the number called in each bulk (513 899 and 792 617). After running the amino acid change and applying the chi‐square test, 1444 SNPs potentially linked to caffeine were identified, of which a number of SNPs were on the same CDS resulting in 1086 CDS. Blast2GO outputs showed only 189 genes containing Kyoto Encyclopaedia of Genes and Genomes (KEGG) pathways in which 70 pathways and 80 enzymes were unique (Table S6).

**Table 3:** Sequencing statistics of two extreme bulks for low/high caffeine and statistics of SNPs discovery and analysis

Parameters	Low‐caffeine bulk	High‐caffeine bulk
No. of individuals	18	18
Average content (% dmb)	1.03	1.48
Total of reads after trimmed (#)	230 140 744	324 14 616
Average coverage (×)	28	40
GC content (%)	36	36
Average Phred score	37	37
Average length after trim (bp)	147	147
No. of variant called in each bulk against the reference	513 899	792 617
No. of variant between two bulks	18 469	
No. of nonsynonymous between two bulks	1444	
No. of CDS	1086	
No. of genes with KEGG pathways	189	
No. of unique KEGG pathways	70	
No. of unique enzymes	80	

#### KEGG pathway‐based analysis for detection of trait‐associated SNPs for caffeine

**Figure 1:**
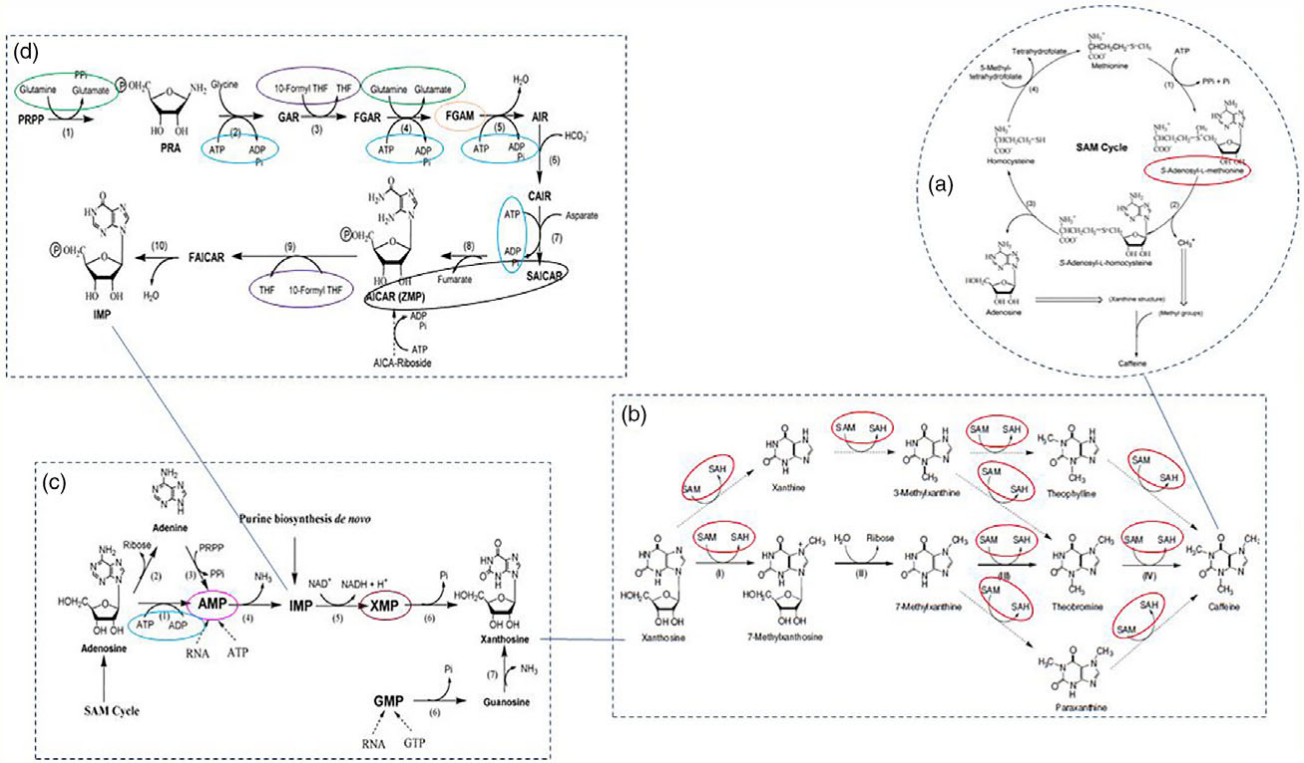
Substrates involved in caffeine biosynthesis pathways and its other related pathways catalysed by enzymes that were encoded by genes carrying the TAVs identified from this study. (a) The SAM (S‐adenosyl‐Lmethionine) cycle (the activated methyl cycle) in plants (adapted from Ashihara and Suzuki, 2004); (b) the biosynthetic pathways of caffeine from xanthosine (adapted from Ashihara *et al*., 2011b); (c) the ‘provider pathways’ for xanthosine synthesis in purine alkaloid forming plants (adapted from Ashihara and Suzuki, 2004); (d) *de novo* biosynthetic pathway of IMP in plants (adapted from Ashihara and Suzuki, 2004); circles: location of substrates/precursors, which were formed in the KEGG pathways catalysed by enzymes encoded by genes carrying the TAVs identified from this study. Circles of the same colour indicated the alternative locations of the same substrate.

Among the 70 KEGG pathways recorded, purine metabolism was the most common pathway with 43 sequences and eight enzymes involved, followed by thiamine metabolism with 29 sequences. The other pathways with large numbers of SNPs were biosynthesis of antibiotics (15 sequences), starch and sucrose metabolism (15 sequences) and pyrimidine metabolism (nine sequences) (Table S6). The 70 KEGG pathways were thoroughly examined to record the processes and enzymes linking to caffeine biosynthesis and narrowed down to seven pathways with ten enzymes present in 65 sequences (66 SNPs) linked to caffeine biosynthesis through eight substrates or precursors that entered the caffeine pathway (Table 4 and Figure 1).

**Table 4:** Substrates, pathways and enzymes involved in caffeine biosynthesis pathway associated with the TAVs identified

Substrates	Pathway	EC	Metabolism^a^	No. of seq^b^
SAM	Cysteine and methionine metabolism	EC:2.1.1.37‐(cytosine‐5‐)‐methyltransferase	A	1
XMP	Purine metabolism	EC:6.3.5.2‐synthase (glutamine‐hydrolysing)	C	2
AMP	Purine metabolism	EC:4.3.2.2‐lyase	A	1
AICAR–SAICAR	Purine metabolism	EC:4.3.2.2‐lyase	A	1
FGAM	Purine metabolism	EC:6.3.5.3‐synthase	A	2
10‐Formyl‐THF	One carbon pool by folate	EC:3.5.1.10‐deformylase	A	1
Glutamate	Arginine biosynthesis	EC:2.3.1.1‐N‐acetyltransferase	C	1^c^
	Arginine and proline metabolism	EC:2.7.2.11‐5‐kinase	C	1
	Carbapenem biosynthesis	EC:2.7.2.11‐5‐kinase	C	1
	Glutathione metabolism	EC:2.5.1.18‐transferase	A	1
ATP–ADP	Purine metabolism	EC:3.6.1.15‐phosphatase	A	29^d^
		EC:3.6.1.3‐adenylpyrophosphatase	A	24^d^
	7 pathways	10 enzymes		65

a Type of metabolism: C: Catabolism (breakdown) of the substrate; A: Anabolism (synthesis) of the substrate.

b Sequences where TAVs are located.

c One sequence with two SNPs.

d The 24 was duplicated with 29; Number in the same colour come from the same sequences (CDS).

Of 65 sequences, 39 were unique (CDS) with 40 unique SNPs (Table S7). The average coverage at the location where SNPs were called was very high for the two bulks (28× for B1 and 37× for B2) (Table S4). Similarly, the forward and reverse read balances were 0.30 and 0.32 for B1 and B2, respectively. The average quality was 36 for B1 and 35 for B2.

**Figure 2:**
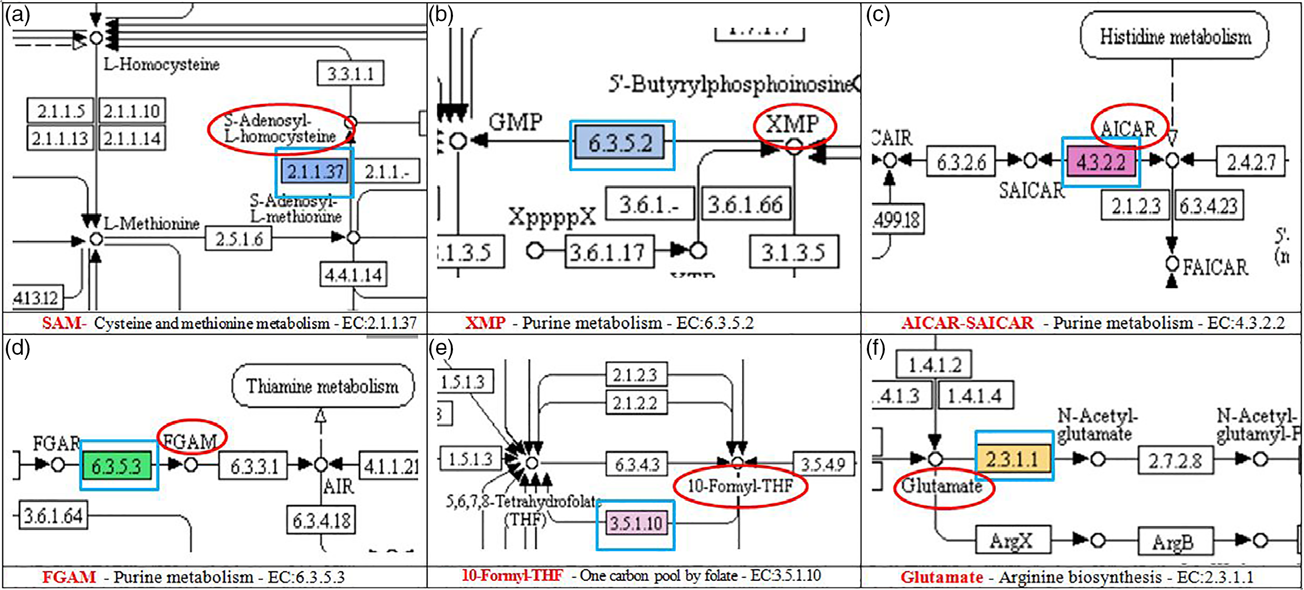
Snapshots of the KEGG pathways (obtained from Blast2GO analysis) at the location where SNPs were associated with enzymes involved in the metabolism of the substrates that entered to the caffeine biosynthesis pathway. Substrates are circled in red; enzymes are highlighted in blue squares.

Among the seven candidate pathways linked to caffeine, the purine metabolism was the most common and generated five substrates (Table 4) entering the caffeine pathway. The ten enzymes [where trait‐associated SNPs (TASs) were located] were involved in three biosynthesis pathways including (i) caffeine with the conversion of SAM, a methyl donor, to *S*‐adenosyl‐l‐homocysteine (SAH) [EC:2.1.1.37‐(cytosine‐5‐)‐methyltransferase in cysteine and methionine metabolism] (Figures 1 and 2a); (ii) xanthosine, the initial purine compound in the caffeine biosynthesis pathway, acting as a substrate for the methyl group donated by SAM (Ashihara and Crozier, 2001) (Figure 1) with the formation of XMP and AMP (EC:6.3.5.2‐synthase (glutamine‐hydrolysing) and EC:4.3.2.2‐lyase from purine metabolism (Figure 2b); (iii) IMP—a precursor of xanthosine (Figure 1) with the formation of AICAR‐SAICAR, FGAM, 10‐formyl‐THF, glutamate and ATP–ADP (Figures 1 and 2c–f).

## Discussion

### Input data for genome assembly

Phred score is an indicator of the read quality, Phred score of 30 indicates a probability of 0.1% of a wrong base call (Dohm *et al*., 2008). In this study, the Phred score of 39 for both paired‐end and MP indicated good‐quality reads. GC content in K7 arabica variety is relatively low (37%–40%). The GC content difference is a primary factor for nonrandom sequencing depth distribution (Li *et al*., 2010a). Sequencing irregularities due to sequence‐dependent coverage biases and nonuniform error rates will cause unexpectedly low‐coverage regions (e.g. Illumina sequencers have lower coverage in low‐GC regions) and consequently more gaps in an assembly (Schatz *et al*., 2010).

The coverage of Illumina data of 137× (both PE and MP) should be sufficient for assembly (Table S1) based on the literature. Ekblom and Wolf (2014) suggested that the total read coverage should be more than 100× for large and complex genomes. Illumina input data are short reads, which may result in fragmented assembly (Schatz *et al*., 2010), but this is complemented by additional long reads of PacBio albeit with a low coverage (6.0×). According to Faino and Thomma (2014), a combination of different sequencing platforms including Illumina reads at 30× coverage (PE reads of 100–150 bp derived from a library with 500‐bp inserts and MP reads of 50–100 bp from a 5‐kb insert library) and 5–10× coverage using SMRT sequencing of a 20 kb insert would give optimal assembly statistics.

### Genome assembly

#### *SOAPdenovo2* outperformed other assemblers when using Illumina data for assembly

Initial genome assembly using CLC GWB proved unsatisfactory with the current data set. The introduction of ABySS and its algorithm as well as stages of assembly was reported by Simpson *et al*. (2009). Although a number of previous studies found that ABySS assembler generated some of the best assembly statistics when only PE Illumina reads were used for different bacteria (Boetzer *et al*., 2011; Boisvert *et al*., 2010; Utturkar *et al*., 2014) or 20 Gb white spruce (Birol *et al*., 2013), the present study showed that ABySS assembler seems to fail in giving sufficient results because the total genome length without gaps only covered a low percentage of estimated genome of arabica (38.5%) and low scaffold N50 (8987 bp).

PLATANUS, another assembler for short reads recently developed by Kajitani *et al*. (2014) to assemble heterogeneous diploid plant genomes, was used to seek improvement. The number of scaffolds and scaffold N50 in PLATANUS were slightly better than others; however, total genome length was small, only covering 20.6% genome size (Table S2). Patel *et al*. (2015) compared PLATANUS with ALLPATHS‐LG in genome assembly of highly heterozygous grape species and found PLATANUS was better. However, Chin *et al*. (2016) found genome assembly results using PLATANUS were poor for *Arabidopsis*, grape and fungus. PLATANUS was also tested on yeasts, fungus and *Arabidopsis* using PE and MP reads (Pryszcz and Gabaldon, 2016). Results showed that PLATANUS deals well over the full spectrum of loss of heterozygosity, but it fails at divergences above 10% and more fragmented than those produced by another new assembler called Redundans (Pryszcz and Gabaldon, 2016). When using additional Illumina MP reads and long reads from PacBio, the neem tree genome quality was improved with PLATANUS, and PLATANUS performed better than SOAP*denovo*2 in regard to assembly statistics for this heterozygous genome (Krishnan *et al*., 2016).

SOAP*denovo*2 outperformed other assemblers with the highest genome assembled covering 62.6% of estimated genome size of 1.3 Gb, and the scaffold N50 was slightly lower than PLATANUS (Table S2). SOAP*denovo*2 performed well on short reads (Zhang *et al*., 2011). SOAP was first developed by Li *et al*. (2010b), and it was compared with other assemblers (ABySS, Velvet, EULER‐SR, SSAKE and Edena) on human genome data. Results showed SOAP obtained better N50 contig, higher genome coverage and shorter running time, higher assembly accuracy; however, it requires a much higher peak memory usage (Li *et al*., 2010b). SOAP*denovo*2 was then designed for certain improvements (Luo *et al*., 2012). According to Simpson and Durbin (2012), SOAP*denovo*2 gave N50 smaller than ABySS and lower assembly completeness, but higher assembly accuracy on nematode genome assembly. In this study, SOAP*denovo*2 outperformed other software and was applied to next steps for assembly improvement.

#### A draft genome assembled using both Illumina and PacBio reads

GapCloser is a module in SOAP, which attempts to resolve gaps in genome assemblies (Luo *et al*., 2012). SSPACE and SOAP*denovo*2 are fast to run on all data sets and perform well with respect to correct versus incorrect joins. SSPACE comfortably has the most citations of any of the scaffolding tools and is also the easiest of the tools to install and run. Combination of SOAP*denovo*2 with SSPACE generally outperformed other scaffolders (Hunt *et al*., 2014).

PacBio long‐read data have emerged as a way of filling N regions in scaffolds (English *et al*., 2012). According to Ekblom and Wolf (2014), the coverage and sequencing platform selection are specific to each project, which requires basic knowledge on genome size, sequencing error rates, repeat content and the degree of genome duplications to make decisions. If the genome has a high repeat content or a high degree of duplications, a larger amount of long‐insert data is needed for correct assembly (Ekblom and Wolf, 2014). In this study, low‐coverage PacBio reads were used for scaffolding using SSPACE LongReads. The output of this step was subjected to GAPCloser again using Illumina (PE & MP) reads. The PBJelly method (English *et al*., 2012) is a gap‐filling approach that takes scaffolds generated by SSPACE LongReads and fills scaffold gaps using long reads. PBJelly2 can use long reads to correct erroneously scaffolded contigs and to close gaps, provided that a quality score is associated with each read (English *et al*., 2012). The addition of PacBio data, even with low coverage, showed improvement in terms of basic assembly metrics (Table S4). With the addition of only 10× coverage of PacBio long reads (20‐kb library) to the Illumina assembly, genome assembly statistics for fungus showed the contig N50 length increased up to 25 times while the gaps reduced approximately three times (Faino and Thomma, 2014). In the current study, scaffold N50 was only double with the addition of 6× PacBio long reads. The total scaffold length was 11% higher than estimated genome size of arabica of 1.3 Gb. This expansion phenomenon was also observed in several crops such as grapevine genome assembly (4.8%) (Jaillon *et al*., 2007), walnut genome assembly (10%–24%) (Martınez‐Garcıa *et al*., 2016) or in pineapple (32%–48%) after the first and second draft assembly (Redwan *et al*., 2016). One reason could be that the high ploidy level or the high heterozygosity rate in arabica made the genome assemblers assume that the genome is diploid (Redwan *et al*., 2016). Another reason could be that SOAP*denovo*2 overestimated gap sizes during scaffolding. The gap (N content) seems to be slightly high, but as several rounds of scaffolding were performed, this issue could be explained. It is most likely that these gaps are complex regions that the assembler could not resolve, or there was not enough sequence coverage, which could be overcome by adding more sequence data.

Genome assembly for ploidy crop such as arabica is faced with certain challenges including genome size, repeat content, paralogy and heterozygosity (Michael and VanBuren, 2015). In addition, the low‐coverage input data in the present study made the arabica genome assembly even more challenging. Arabica has the genome size estimated at 1.3 Gb (Kochko *et al*., 2010), which is fairly large compared to other common genome species such as Arabidopsis, rice, grape and sorghum (Michael and VanBuren, 2015). A large and complex genome has large repetitive elements and covers a large fraction of the genome resulting in ambiguities in the scaffolding step (Madoui *et al*., 2016). A tetraploid species such as arabica has different ‘copies’, which tend to be less similar, while the algorithms and software developed for assembly were mainly developed for haploid or diploid genomes that may lead to the risk of information loss when using it for genome assembly in polyploid crops (Margarido and Heckerman, 2015). As arabica is an allotetraploid crop, it is expected to show heterozygosity conditioned by two different alleles derived from two different progenitors (Mishra *et al*., 2011). Paralogous regions and heterozygous sites create ‘bubbles’ during genome assembly where two or more regions that are highly similar assemble together, and the adjacent dissimilar regions assemble separately but eventually merge again (Michael and VanBuren, 2015) make it hard for assembly. It is suggested that coverage for finished assemblies was 50× for long reads (Koren and Phillippy, 2015) while there was only 6× coverage for PacBio data in the present study.

In summary, the K7 arabica genome assembled was higher than estimated for the arabica genome. After being optimized with many steps using different assemblers, scaffolders and gap closers and with different sequencing reads, the gap length was still large and accounts for 21% of estimated genome. This is, however, an encouraging outcome given the challenges in Arabica genome assembly such as high ploidy level, heterozygosity, low coverage and fairly large genome. Fragmented genome assembly was also reported for *C. canephora* (13 345 scaffolds) (Denoeud *et al*., 2014) and other species. In date palm with half the genome size of arabica, the draft genome was still in 57 277 scaffolds and N50 scaffold of 30 480 bp (Al‐Dous *et al*., 2011). Similarly, walnut with half the genome size of arabica, the draft genome was with 186 636 scaffolds (Martınez‐Garcıa *et al*., 2016). For larger genome size like rubber (2.15 Gb haploid genome), the genome assembly was with 608 017 scaffolds and N50 scaffold of 2972 bp (Rahman *et al*., 2013). Even the Arabidopsis genome, which is arguably the best‐assembled plant genome, is still in 102 contigs with a total gap length of at least 185 644 bp (Michael and VanBuren, 2015).

### Validation of draft genome

To assess the genome completeness and to detect errors in the assembly, Illumina short reads (PE) were aligned back to the assembly outcomes using BWA (Li and Durbin, 2009). The genome quality assessment was evaluated by remapping short reads to the final draft using BWA. Results showed high percentage of reads mapped back to the draft suggesting that most of the reads were incorporated into the genome, and thus, most of the genomes were assembled.

Validation of the assembly or gene space completeness can be based on the genomic resources available for coffee such as *C. canephora* genes or ESTs database of *C. arabica* (187 739 ESTs from NCBI) aligned to *C. arabica* draft genome using GMAP. In this study, *C. canephora* CDS sequences and *C. arabica* (K7) PacBio transcriptome data were used to map back to the draft genome. High percentage of the CDS and transcriptome sequences were mapped to *C. arabica* draft genome (99%) with high identity (≥90%) and query coverage (90%) indicating the completeness of the draft genome (Table 2).

Another approach to assess the completeness of genome assembly was running CEGMA (Core Eukaryotic Genes Mapping Approach) (Parra *et al*., 2007, 2009) or BUSCO (benchmarking universal single‐copy orthologs plant conserved genes) (Simao *et al*., 2015) in order to identify putative core eukaryotic genes (CEGs) and universal single‐copy orthologs (USCOs) in the assembly. CEGMA has been replaced with BUSCO, which is newly developed and more comprehensive than CEGMA (Simao *et al*., 2015) and was used in this study. BUSCO analysis against a plant‐specific database of 956 genes identified 858 (89%) complete BUSCOs (Table 2). The high percentage of BUSCOs mapped to the draft genome indicated the high completeness of the assembly. According to Simao *et al*. (2015), the high amount of duplicated complete BUSCOs indicated the erroneous assembly of haplotypes. However, Lee *et al*. (2016) proved that this links to genome duplication or recent hybridization between seagrass species, which is also the case of arabica (Lashermes *et al*., 1999; Tesfaye *et al*., 2007). Sayadi *et al*. (2016) also stated that this may represent allelic variation (heterozygosity) in the sample used to construct the assembly, gene duplication and/or mechanisms such as alternative splicing. Using different approaches of genome completion assessment indicted the completion of the genome assembly. The draft genome was then subjected to annotation to facilitate the downstream analysis.

### Annotation of draft genome

The number of genes in the first annotation is lower than expected as it is close to the number of one of its ancestor *C. canephora* (25 574 protein‐coding genes) (Denoeud *et al*., 2014). The low number of genes is likely because the genomic database used was limited. In the final annotation, when using other public databases as reference, especially with tomato—the closest plant species to coffee—the number of genes was almost four times higher than its double haploid progenitor. This number may be explained by the ‘true’ tetraploid nature of K7, which is four times larger in genome size compared to the double haploid canephora. Compared to other closely related plant species such as grape (30 425 genes), tomato (34 771 genes) and potato (35 004 genes) (Tomato‐Genome‐Consortium, 2012), K7 arabica is almost three times higher.

Functional annotation probably reveals more insight into the K7 genome, especially when comparing with one of its progenitors—*C. canephora*.

### SNPs for caffeine discovered using the draft genome as reference

#### Mapping and SNPs identification

The average coverage of two bulks of 28× and 40× met the recommended depth for pooled sequencing, which should be at least equal to or higher than the number of individuals in a pool (Magwene *et al*., 2011). The Phred score (an indicator of the read quality) was 37, which is higher than the recommended Phred score of 30 (Dohm *et al*., 2008), and the GC content of 36% was in the good range (Li *et al*., 2010a).

As the average coverage of two bulks of low and high caffeine is 28× and 40×, setting for minimum coverage therefore was at 20 x. For tetraploid species such as *C. arabica*, identification of high confidence SNPs is challenging as one locus potentially has up to four alleles (i.e. allele frequencies could be 25%, 50%, 75% or 100%) (Castle *et al*., 2014). However, in pool sequencing, the allele frequency will not follow the theoretical scenarios due to possible experimental noise during sampling, chemical analysis and DNA mixture, which might result in unbalance representation of individuals in the pool. Therefore, in the present study, the minimum count or minimum of reads to be called as a variant was set at 20% (or 4 per 20 reads).

The application of amino acid changes tool and chi‐square test is very fundamental to narrow down the number of SNPs to work with. While the amino acid changes tool helps to identify the nonsynonymous SNPs (caused by changing amino acid), the chi‐square test is to ensure the difference between two bulks is significant. In addition, the ‘If command’ tool in Excel (allele one or two in bulk 1 must be ≥50%, while the corresponding allele in the other bulk must be ≤50%) was applied to select the nonsynonymous SNPs between the two bulks and not between each bulk and the reference. The final SNPs were checked manually using the mapping and variant calling files in the form of tracks to gain confidence. The number of highly confident SNPs of 1444 on 1086 CDS is a reasonable number to handle for downstream functional analysis. The detection of significant SNPs between groups of extreme phenotypes for caffeine indicates the possible presence of major genes for this trait. These would show a diploid mode of inheritance in *C. arabica* despite it being an allotetraploid species (Krug and Mendes, 1940; Lashermes *et al*., 2000; Teixeira‐Cabral *et al*., 2004).

#### KEGG pathway‐based analysis for detection of TAS for caffeine

The high average coverage at the location where SNPs were called indicated the high quality and confidence of these SNPs. The confidence was supported with the high forward/reverse balance in reads and the average base quality score as these are a reflection of read quality used in SNP detection.

That five of eight substrates that link to the caffeine pathway were from purine metabolism, and this is not surprising as xanthosine is synthesized via purine. Caffeine is one of the purine alkaloids, and biosynthetic pathways to these purine alkaloids from purine nucleotides in tea and coffee plants have been proposed (Ashihara *et al*., 1996).

Among 41 unique SNPs that potentially link to) the caffeine biosynthesis pathway, the most noticeable one is the SNP associated with cytosine‐5‐methyltransferase (EC 2.1.2.37) participating in the conversion of SAM to SAH in the SAM cycle (Figure 2a) (Ashihara and Suzuki, 2004). The SNP was located in contig 35 775 at the 294 310‐bp position. The low‐caffeine bulk has more G‐allele and less C‐allele than the high‐caffeine bulk. As explained by Guo *et al*. (1996), the differences in allele dosage may result in differences in the RNA levels of a particular allele and in phenotypic differences. The change in more G in B1 while more C in B2 led to the change in amino acid from alanine to proline (Table S7). This enzyme would have an influence on the formation of the methyl group and thus might influence the synthesis of caffeine (Figure 1a).

The five SNPs associated with four enzymes participating in purine metabolism converting SAICAR to AICAR and FGAR to FGAM or forming XMP and AMP were located in five contigs (Table S7). The enzyme EC:4.3.2.2‐lyase can convert SAICAR to AICAR or vice versa (Table 4 and Figure 2c) and also converts adenylosuccinate to AMP while enzyme EC:6.3.5.2‐synthase (glutamine‐hydrolysing) converts XMP to GMP (Figure 2b) and EC:6.3.5.3‐synthase converts FGAR to FGAM (Figure 2d). All these substrates enter the xanthosine biosynthesis pathway (Figure 1c and d). SNP alleles at these locations were all heterozygous and different in frequency resulting in a change in amino acid. The presence of more or less of the specific SNP alleles in each individual may have contributed to the synthesis and conversion of the aforementioned substrates causing the difference in the concentration of caffeine between the two extreme groups.

The conversion of 10‐Formyl‐THF to 5,6,7,8‐tetrahydrofolate (THF) is one of the reactions in the synthesis pathway of IMP (Figure 1d), which is catalysed by the enzyme EC:3.5.1.10‐deformylase (Table 4). The SNP (contig 3241 at location 30 206) linked to this enzyme was a C‐allele only (100%) in the high‐caffeine bulk, while the low‐caffeine bulk has C‐allele (71%) and A‐allele (29%) resulting in a change in amino acid (arginine only in the high bulk, both arginine and isoleucine in the low bulk) (Table S7).

Glutamine is converted to glutamate in the IMP biosynthesis pathway which eventually produces xanthosine—the first substrate in the caffeine synthesis pathway (Figure 1) (Ashihara *et al*., 2011b). There were four SNPs associated with three enzymes participating in the metabolism of glutamate (Table 4), and all of them are heterozygous and different in frequency resulting in the change in amino acids (Table S7).

ATP (adenosine triphosphate) and ADP (adenosine diphosphate) are organic nucleotide molecules. ATP is converted to ADP in the cells of plants and animals when energy is required to power processes in the cell with the energy released. Energy is also released when a phosphate is removed from ADP to form adenosine monophosphate (AMP). Although AMP is a substrate that participate in the synthesis pathway of xanthosine, ATP and ADP are generic substrates for chemical reactions so their TASs will not be discussed further. Instead, one SNP associated with two enzymes (EC:3.6.1.3‐adenylpyrophosphatase and EC:3.6.1.15‐phosphatase) in purine metabolism pathways that convert ATP to ADP interacting with the xanthosine synthetic pathways (Figure 1 and Table 4) was considered. This significant SNP was a homozygous allele in the high‐caffeine bulk (100% C‐allele), while heterozygous alleles (C‐ and A‐allele of 48% and 52%, respectively) were identified in the low‐caffeine bulk (Table S7). The presence of the A‐allele in the low bulk probably effects the conversion from ATP to ADP resulting in less caffeine being synthesized.

## Conclusions

The discovery of the genetic control of caffeine was advanced using a draft genome assembly to analyse sequences of phenotypic bulks. Using the hybrid approach in assembly with several assemblers, gap fillers and scaffolders resulted in 76 409 scaffolds with scaffold N50 of 54 544 bp. The total scaffold length was 1448 Mb, which is 11% higher than the estimated arabica genome (1.3 Gb). This expansion could be attributable to the effect of ploidy and heterozygosity levels in arabica, which could not be resolved using the existing genome assemblers. Development and deployment of software that is suitable for a highly heterozygous genomes or polyploidy combined with longer read sequencing technology (e.g. Nanopore) may help to reduce the expansion and fragmentation of the sequenced genome. Altogether, 99 829 gene models were annotated when using public database as reference, which is four times higher than that of the double haploid canephora. Currently, there are several groups working on a more complete arabica genome assembly using different genotypes, sequencing platforms, assemblers and scaffolders (Gaitan *et al*., 2015; Morgante *et al*., 2015; Strickler, 2015; Yepes *et al*., 2016). Although the draft genome used here had gaps and was incomplete, it helped to detect a significant number of TASs for caffeine, which may eventually have an important impact on coffee genetics and breeding for this trait. The large numbers of genes interacting to determine the caffeine content of the bean were revealed.

## Experimental procedures

### DNA extraction and pooling for SNP detection

From a diverse population of 232 arabica accessions as described in Tran *et al*. (2017), two extreme phenotypic groups for caffeine were selected such that each contained 18 individuals. Leaf samples were collected from the germplasm plantation at CATIE, Costa Rica. DNA extraction was performed following the method described by Healey *et al*. (2014) with a slight modification using of 2‐mercaptoethanol (0.3%) and PVPP (2%) to reduce the effect of phenolic compounds. The concentration was then standardized and diluted to include an equal amount of DNA for all individual DNA samples. The 18 samples in each extreme phenotypic group were finally mixed to form a DNA bulk, resulting in two DNA bulks of low caffeine (B1) and high caffeine (B2). The DNA quality and quantity of each bulk were then checked using Nanodrop, agarose gel and Qubit meter before sequencing.

### Sequencing and mapping

DNA samples were sequenced as 2 indexed PCR‐free libraries, using a HiSeq 2000 (v4) flow cell of an Illumina platform (Queensland Brain Institute (QBI), University of Queensland).

Paired‐end reads with insert sizes of 150 bp from four DNA bulks were imported to CLC Genomics Workbench Version 10.0 (CLC Bio, www.clcbio.com). The draft genome was also imported to CLC as a standard import and used as a reference for mapping using a length fraction (LF) and similarity fraction (SF) of 1.0 and 0.8.

### Identification of trait‐associated SNPs

The stand‐alone mapping file was used for variant calling. Variants were called using the ‘Basic variant detection’ tool with ploidy level of 4. To ensure that the SNPs identified were of high quality, the SNPs were selected as follows: (i) minimum coverage of at least 20 and maximum coverage of 1000, (ii) broken pairs and nonspecific matches were removed, (iii) minimum of 4 reads or 20% to be called as a variant, (iv) base quality filter minimum central quality of 20, neighbourhood radius of 5, minimum neighbourhood quality of 15 and (v) read direction frequency of 20%.

The tool ‘Identify known mutations from sample mappings’ was used to identify variants between two bulks, followed by the tool ‘Amino acid changes’ to obtain the nonsynonymous SNPs. The chi‐square test was applied to test for significant differences between the two extreme bulks.

The final set of nonsynonymous SNPs was extracted from the annotation track to obtain CDS sequences and imported into Blast2GO (version 4.0.7) (Conesa *et al*., 2005) so as to obtain much information on the functional annotation and biological role of these SNPs. Output data of all KEGG pathways were examined thoroughly to identify those involved in caffeine biosynthetic pathways.

### DNA extraction and genome sequencing

A leaf sample of Arabica variety—K7 (Omondi *et al*., 2016)—was collected from Green Cauldron, 330 Federal Rd, Federal NSW 2480, in October 2014. K7 is a Kenyan selection of French Mission Bourbon selected in Kenya based on cupping trials. In the study of Tran (2005), K7 was clustered with the Typica group—one of the two distinct botanical varieties within *C. arabica*. Historical data indicate that Typica originated from a single plant from Indonesia, which was subsequently cultivated in the Amsterdam botanical garden in the early 18th century. This indicates that K7 is inherently highly homozygous. DNA extraction was performed using the method described above. The DNA from several extractions was mixed and precipitated to obtain an adequate concentration of DNA required for Illumina and PacBio sequencing. DNA quality and quantity were assessed using a Nanodrop spectrophotometer (Thermo Fisher Scientific, Waltham, MA) and agarose gel electrophoresis (Bio‐Rad Laboratories, Hercules, CA).

#### Illumina sequencing

At least 25 μg of total DNA was dissolved in TE‐Buffer (Tris‐EDTA, 10 mm Tris‐HCl, 1 mm disodium EDTA, pH 8.0) and used to prepare sequencing libraries. Sequencing was performed using an Illumina HiSeq 2000 with three libraries including (i) C‐CofK7IL for TruSeq PCR‐free Library HiSeq 2× 100 bp paired‐end sequencing using adapter 04 with an insert size of 350 bp, concentration of 355 ng/μL with volume of 60 μL to make a total of 21 300 ng of DNA; (ii) C‐CofK7IL3 for Nextera 3 kb MP (indexed) Library using adapter 13 and then HiSeq 2× 100 bp paired‐end sequencing, concentration of 447 ng/μL with volume of 90 μL to make a total of 40 230 ng DNA; (iii) C‐CofK7IL8 for Nextera 8 kb MP (indexed) Library using adapter 18 and then HiSeq 2× 100 bp paired‐end sequencing, concentration of 422 ng/μL with volume of 90 μL to make a total 37 980 ng of DNA.

Gel portions for the 3 and 8 kb bands cut from the gel during library preparation were estimated to have an average size of 5.3 and 10.4 kb, respectively, using a Bioanalyzer (Agilent Technologies, Santa Clara, CA). The two Nextera MP Libraries were run together in one lane and demultiplexed evenly with 49% of reads identified for each library. The single TruSeq PCR‐Free library was run in a lane of its own.

#### PacBio sequencing

A sample at a concentration of 303 ng/μL (Qubit QC, Thermo Fisher Scientific, Waltham, MA) and total DNA of 31.8 μg were sequenced in 15 SMRT (Single Molecule Real‐Time) cells using P6‐C4 chemistry (20 KB protocol) resulting in 927 726 reads with an N50 read length of 13 127 bp and a mean read length of 8312 bp.

### Genome assembly

#### Assembly of PE and MP Illumina data

Initially, assemblies of Illumina PE and MP reads were generated using CLC Genomics Workbench, version 9.5 (CLC Bio, www.clcbio.com). The same data set was assembled with alternative assembly algorithms such as ABySS (Simpson *et al*., 2009), PLATANUS (Kajitani *et al*., 2014) and SOAP*denovo*2 (Luo *et al*., 2012). SOAP*denovo*2 resulted in improved assembly statistics and was subjected to GAPCloser (a module in SOAP*denovo*2), then SSPACE Standard (Boetzer *et al*., 2011) for scaffolding, then GAPCloser again using Illumina PE reads.

#### Assembly of PacBio data

The final Illumina assembly was further scaffolded with PacBio long reads using SSPACE‐LR (LongReads) (Boetzer and Pirovano, 2014) for scaffolding, followed by GAPCloser using Illumina reads and finally gap filled with PBJelly2 (English *et al*., 2012) using PacBio long reads.

The assembly generated from SSPACE‐LR was gap filled with GAPCloser (Illumina Data) and PBJelly2 (PacBio long reads). PBJelly2 software is used to fill gaps in the assembly using PacBio long reads. The program is designed to handle PacBio data taking its error model into consideration. It uses a PacBio read data‐specific aligner called BLASR (Chaisson and Tesler, 2012) to map PacBio reads to the assembly and attempt to replace Ns with A, C, G or T. The final output from PBJelly2 was the first draft version of the genome.

### Validation of genome assembly

Validation of assembly was assessed using three different approaches:

1. PE Illumina reads were remapped to detect errors in the assembly using BWA (Li and Durbin, 2009).
2. Available coffee genomic resources such as *C. canephora* CDS (coding DNA sequences) sequences (http://coffee-genome.org/coffeacanephora) and *C. arabica* (K7) PacBio transcriptome data (not published) were used to map back to the draft genome using GMAP (Wu and Watanabe, 2005).
3. The BUSCO (benchmarking universal single‐copy ortholog) (Simao *et al*., 2015) strategy was used to test the completeness of the genome assembly and gene space using the plant‐specific profile. This approach makes use of single‐copy genes expected to be present in plants (956 genes).

### Genome annotation

Final genome assembly was repeat‐masked using REPEATMODELER (Smit and Hubley, 2008) and REPEATMASKER (Smit *et al*., 1996). MAKER‐P (Campbell *et al*., 2014) was run on the repeat‐masked genome with SNAP (Korf, 2004) and AUGUSTUS (Stanke and Morgenstern, 2005). The gene prediction programs, SNAP and AUGUSTUS, used *Arabidopsis thaliana* HMM (Hidden Markov Model) and tomato, respectively. ESTs of *C. arabica* (http://www.ncbi.nlm.nih.gov/nucest) and CDSs of *C. canephora* (http://coffee-genome.org/coffeacanephora) and 96 521 in‐house *C. arabica* (K7) PacBio transcriptome sequences were used as evidence to guide the annotation process.

## Conflict of interest

The authors declare no conflict of interests.

## Supporting information


**Table S1** Parameters of Illumina data and PacBio data.
**Table S2** Assembly statistics among different assemblers with Illumina sequencing reads.
**Table S3** Assembly improvement using GAPCloser and Scaffolders with Illumina sequencing reads.
**Table S4** Assembly improvement using GapClosers and Scaffolders with PacBio longreads.
**Table S5** Statistics of genome annotation.

**Table S6a** 10 most common pathways.
**Table S6b** 80 enzymes recorded from 1444 TAVs.
**Table S6c** 79 pathways recorded from 1444 TAVs.
**Table S7** Detailed information of 66 SNPs associated with caffeine detected by KEGG pathway analysis.
