# Supplementary material for: Use of a draft genome of coffee (Coffea arabica) to identify SNPs associated with caffeine content
Source: Plant Biotechnol J. 2018 Apr 13;16(10):1756–66. doi: 10.1111/pbi.12912 (PMC6131422; doi:10.1111/pbi.12912)
Supplement: Supplementary file 1 — Table S1 Parameters of Illumina data and PacBio data. Table S2 Assembly statistics among different assemblers with Illumina sequencing reads. Table S3 Assembly improvement using GAPCloser and Scaffolders with Illumina sequencing reads. Table S4 Assembly improvement using GapClosers and Scaffolders with PacBio longreads. Table S5 Statistics of genome annotation. [file PBI-16-1756-s001.docx]

**Supplement**

Table S1: Parameters of Illumina data and PacBio data

| **Platform** | **Library** | **Insert size (bp)** | **Read length (bp)** | **No of reads** | **Coverage (*)** | **GC content** | **Ave. Phred score** |
| --- | --- | --- | --- | --- | --- | --- | --- |
| Illumina | Paired end | 350 | 100 | 385.10^6^ | 64 x | 37% | 39 |
|  | Mate pair | 3 Kb | 100 | 220.10^6^ | 36 x | 40% | 39 |
|  |  | 8 Kb | 100 | 223.10^6^ | 37 x | 39% | 39 |
| PacBio | SMRT (15) | - No of bases: 7,688,079,960  - N50 read length: 8,180 b  - Mean read length: 5,579 b | | 1,377,852 | 6 x | 37% |  |

(*) Based on a genome size estimate of 1.3 Gbp

Table S2 Assembly statistics among different assemblers with Illumina sequencing reads

| **Parameters** | **CLC** ^(1)^ | **ABySS** ^(2)^ | **PLATANUS** ^(3)^ | **SOAP denovo** ^(4)^ |
| --- | --- | --- | --- | --- |
| **Contig metrics** | | | | |
| # contig |  | 628,389 | 62,453 | 276,322 |
| Contig N50 |  | 1,188 | 3,627 | 6,284 |
| Ave contig size |  | 798.3 | 2,631.1 | 2,944.1 |
| **Scaffold metrics** | | | | |
| # scaffold | 189,330 | 139,505 | 101,960 | 120,578 |
| Scaffold N50 | 5,490 | 8,987 | 17,991 | 16,694 |
| Min scaffold size | 985 | 1,000 | 1000 | 1000 |
| Max scaffold size | 105,805 | 218,433 | 501,363 | 176,350 |
| Ave scaffold size incl. gaps | 4,230 | 4,265.7 | 4,654.4 | 8,154.9 |
| Ave scaffold size w/o gaps | 1,263 | 3,596.0 | 4,295.5 | 6,746.9 |
| Genome incl. gaps * | 800,884,967 | 595,091,719 | 290,679,196 | 983,303,576 |
| Genome w/o gaps ** | 604,221,306 | 501,664,397 | 268,267,094 | 813,528,413 |

* Total genome length including gaps; ** Total genome length without gaps; ^(1)^ De novo settings of slow mode, word size, bubble size & minimum contig length of 64-120-1000, and including scaffolding; ^(2)^ k-mer of 80 bp, minimum scaffold size of 1000 bp, scaffolded with MP; ^(3) (4)^ minimum scaffold sizes of 1000 bp.

Table S3 Assembly improvement using GAPCloser and Scaffolders with Illumina sequencing reads

| **Software** | **SOAPdenovo** | **GAPCloser**  **(1^st^)** | **SSPACE (standard)** | **GAPCloser**  **(2^nd^)** |
| --- | --- | --- | --- | --- |
| **Contig metrics** | | | | |
| # contigs | 602,834 | 276,322 | 411,313 | 320,474 |
| Ave. contig size | 1,111 | 2,944 | 2,236 | 3,135 |
| Contig N50 | 2,063 | 6,284 | 6,571 | 8,522 |
| **Scaffold metrics** | | | | |
| # scaffolds | 120,114 | 120,578 | 110,179 | 110,443 |
| Ave. scaffold size incl. gaps | 8,118 | 8,155 | 12,281 | 12,277 |
| Ave. scaffold size w/o gaps | 5,575 | 6,747 | 8,346 | 9,098 |
| Scaffold N50 | 16,569 | 16,694 | 27,147 | 27,310 |
| Max scaffold size | 175,948 | 176,350 | 389,435 | 389,501 |
| Min scaffold size | 1,000 | 1,000 | 1,000 | 1,000 |
| Total genome length incl. gaps | 975,074,759 | 983,303,576 | 1,353,166,521 | 1,355,930,976 |
| Total genome length w/o gaps | 669,693,502 | 813,528,413 | 919,608,986 | 1,004,783,410 |
| **Gap metrics** | | | | |
| Captured gaps | 482,683 | 155,718 | 359,134 | 210,009 |
| Max gap | 27,925 | 27,658 | 27,658 | 27,658 |
| Mean gap | 633 | 1,090 | 1,207 | 1,672 |
| Gap N50 | 3,731 | 4,751 | 4,957 | 5,270 |
| Total gap length | 305,378,544 | 169,774,888 | 433,614,750 | 351,147,504 |

Table S4 Assembly improvement using GapClosers and Scaffolders with PacBio longreads

| **Assembler** | **GAPCloser (Illumina reads)** | **SSPACE-Longsreads**  **(PacBio reads)** | **GAPCloser (Illumina reads)** | **PBJelly2**  **(PacBio reads)** |
| --- | --- | --- | --- | --- |
| **Contig metrics** | | | | |
| # contigs | 320,474 | 320,178 | 283,660 | 265,687 |
| Max contig | 183,259 | 183,259 | 186,627 | 186,701 |
| Mean Contig | 3,135 | 3,148 | 3,778 | 4,393 |
| Contig N50 | 8,522 | 8,592 | 10,133 | 12,184 |
| Contig N90 | 1,392 | 1,400 | 1,732 | 2,110 |
| Total contig length | 1,004,783,410 | 1,007,838,579 | 1,071,793,316 | 1,167,243,096 |
| Assembly GC (%) | 37.00 | 36.63 | 36.71 | 36.76 |
| **Scaffold metrics** | | | | |
| # scaffolds | 110,443 | 76,673 | 76,673 | 76,409 |
| Max scaffold | 389,501 | 755,558 | 757,040 | 769,411 |
| Mean scaffold | 12,277 | 18,292 | 18,314 | 18,954 |
| Scaffold N50 | 27,310 | 52,292 | 52,431 | 54,544 |
| Scaffold N90 | 5,476 | 8,105 | 8,105 | 8,145 |
| Total scaffold length | 1,355,930,976 | 1,402,516,201 | 1,404,209,962 | 1,448,282,977 |
| **Gap metrics** | | | | |
| Captured gaps | 210,031 | 243,505 | 206,987 | 189,278 |
| Max gap | 27,658 | 27,658 | 27,141 | 27,141 |
| Mean gap | 1,672 | 1,621 | 1,606 | 1,485 |
| Gap N50 | 5,270 | 4,844 | 5,050 | 5,282 |
| Total gap length | 351,147,566 | 394,677,622 | 332,416,646 | 281,039,881 |

Table S5: Statistics of genome annotation

| **Type** | **Total number** |
| --- | --- |
| CDS (Median : Mean; Min-Max) | 384,252 (138 : 228; 2 - 9,191) |
| Genes (Median : Mean; Min-Max) | 99,829 (1,523 : 2,612; 49 - 51,554) |
| Intron (Median : Mean; Min-Max) | 292,819 (258 : 590; 5 - 26,764) |
| Start codon | 92,584 |
| Stop codon | 93,413 |
| Transcript | 99,829 |
